# The Adr1 transcription factor directs regulation of the ergosterol pathway and azole resistance in *Candida albicans*

**DOI:** 10.1128/mbio.01807-23

**Published:** 2023-10-04

**Authors:** Manjari Shrivastava, Gaëlle S. Kouyoumdjian, Eftyhios Kirbizakis, Daniel Ruiz, Manon Henry, Antony T. Vincent, Adnane Sellam, Malcolm Whiteway

**Affiliations:** 1 Department of Biology, Concordia University, Montréal, Quebec, Canada; 2 Center for research, Montreal Heart Institute, Montréal, Quebec, Canada; 3 Department of Microbiology, Infectiology and Immunology, Faculty of Medicine, Université de Montréal, Montréal, Quebec, Canada; 4 Department of Animal Sciences, Université Laval, Quebec City, Canada; Universidade de Sao Paulo, Ribeirao Preto, Sao Paulo, Brazil

**Keywords:** rewiring, Adr1, ergosterol, *Candida albicans*, alcohol and fatty acid metabolism

## Abstract

Transcription factors (TFs) play key roles in cellular regulation and are critical in the control of drug resistance in the fungal pathogen *Candida albicans*. We found that activation of the transcription factor C4_02500C_A (Adr1) conferred significant resistance against fluconazole. In *Saccharomyces cerevisiae*, Adr1 is a carbon-source-responsive zinc-finger transcription factor required for transcription of the glucose-repressed gene *ADH1* and of genes required for ethanol, glycerol, and fatty acid utilization. Motif scanning of promoter elements suggests that Adr1 may be rewired in fungi and govern the ergosterol synthesis pathway in *C. albicans*. Because previous studies have identified the zinc-cluster transcription factor Upc2 as a regulator of the ergosterol pathway in both fungi, we examined the relationship between Adr1 and Upc2 in sterol biosynthesis in *C. albicans*. Phenotypic profiles of either *ADR1* or *UPC2* modulation in *C. albicans* showed differential growth in the presence of fluconazole; either *adr1* or *upc2* homozygous deletion results in sensitivity to the drug, while their activation generates a fluconazole-resistant strain. The rewiring from ergosterol synthesis to fatty acid metabolism involved all members of the Adr1 regulon except the alcohol dehydrogenase Adh1, which remains under Adr1 control in both circuits and may have been driven by the lifestyle of *S*. *cerevisiae*, which requires the ability to both tolerate and process high concentrations of ethanol.

**Importance:**

Research often relies on well-studied orthologs within related species, with researchers using a well-studied gene or protein to allow prediction of the function of the ortholog. In the opportunistic pathogen *Candida albicans*, orthologs are usually compared with *Saccharomyces cerevisiae*, and this approach has been very fruitful. Many transcription factors (TFs) do similar jobs in the two species, but many do not, and typically changes in function are driven not by modifications in the structures of the TFs themselves but in the connections between the transcription factors and their regulated genes. This strategy of changing TF function has been termed transcription factor rewiring. In this study, we specifically looked for rewired transcription factors, or *Candida*-specific TFs, that might play a role in drug resistance. We investigated 30 transcription factors that were potentially rewired or were specific to the *Candida* clade. We found that the Adr1 transcription factor conferred resistance to drugs like fluconazole, amphotericin B, and terbinafine when activated. Adr1 is known for fatty acid and glycerol utilization in *Saccharomyces*, but our study reveals that it has been rewired and is connected to ergosterol biosynthesis in *Candida albicans*.

## INTRODUCTION

*Candida albicans* is an opportunistic fungal pathogen that is responsible for a variety of fungal infections in humans. In healthy people, this yeast resides as a commensal in niches such as the gastrointestinal tract, but it can cause mucosal, cutaneous, and systemic infections in immunocompromised individuals (1). The prevalence of resistance to antifungal agents has significantly increased in the past few decades, and this resistance has important implications for mortality, morbidity, and health care in the community (2). The development of new antifungal drugs is challenging, as fungi are eukaryotic organisms that share many basic cellular processes with humans, and this evolutionary relatedness makes the identification of specific targets difficult and increases the likelihood of undesired secondary effects. Consequently, existing antifungals tend to target processes that are divergent between fungi and the human host.

The azole class of antifungals, including fluconazole, targets the ergosterol pathway, inhibiting a step not found in the pathway for the host-specific sterol cholesterol. Azoles are generally effective for the management of *C*. *albicans* infections, but due in part to the fungistatic nature of the drugs, long-term treatment often results in the emergence of azole resistance, ultimately resulting in therapeutic failure (3 – 5). These azole antifungals bind and inhibit the activity of the enzyme lanosterol 14-alpha-demethylase encoded by *ERG11* (6). Apart from azoles, allylamines (which target Erg1), polyenes (which target ergosterol itself), morpholines (which target Erg2), and statins (which target HMG1/2) also target elements of the sterol pathway (7). As many drugs target the *C. albicans* sterol pathway, genetic changes that perturb the pathway can lead to multi-drug resistance (8, 9).

A promising approach for drug development is to identify synergistic targets that can enhance the antifungal effect of currently available drugs (10). Transcription factors (TFs) play a key role in determining how cells function and respond to different environments, and approximately 4% of *C. albicans* genes encode transcription factors (11), making them the single largest family of proteins in the pathogen. TFs in *C. albicans* coordinate critical cellular functions, including biofilm formation (12), drug resistance (13), and the transition from a commensal to a pathogenic lifestyle (14).

Most transcription factors are conserved in that they fall into a limited number of groups of structurally similar proteins, such as the zinc finger, the basic helix loop helix, and the leucine zipper classes. However, evolutionary changes in transcription networks are an important source of diversity across species, driven primarily not by major changes in the structures of the factors themselves but in the connections between the transcription factors and their regulated genes. There are many incidences where researchers have identified structurally equivalent transcription factors regulating different genetic circuits in different organisms (15 – 18); this phenomenon has been called “rewiring.” Studies suggest that this rewiring happens at a relatively constant rate, and for two species that have diverged for 100 million years, only a fraction of transcription factor/target gene combinations will likely have remained conserved (19, 20). *C. albicans* belongs to the same family as *Saccharomyces cerevisiae*, but the two fungi are suggested to have diverged as long ago as 300 million years, allowing for considerable rewiring. While studies of TFs have tended to focus on similarities between these two species, it has been estimated that only 16% of the regulator-target gene connections are preserved between the *C. albicans* and *S*. *cerevisiae* (21).

In our study, we have activated a group of transcription factors for *C. albicans* for which there was limited information and which had the potential to be rewired. Among our tested set of TFs, we found that C4_02500C_A activation gives resistance to several cell-membrane-targeting drugs. This resistance arises because C4_02500C_A is a central regulator of the ergosterol pathway in *C. albicans*. Further analysis shows that this TF is the ortholog of *S. cerevisiae* Adr1 and that the two proteins play distinct cellular roles in the two species.

## RESULTS

### Fusion of different transcription factors to the strong activation domain VP64

In *S. cerevisiae*, the fusion of VP16 to the N terminus of Gal4 resulted in the hyper-activation of Gal4 (22, 23), and the VP64 fusion has been used to successfully activate transcription factors in both plants (24) and animals (25). We have used a similar strategy in *C. albicans*. Fusing a tetrameric version of the VP16 trans-activating domain (VP64) to the DNA-binding domains of different *C. albicans* transcription factors was found to be potent in transcriptional activation (26), so we constructed plasmid CIPACT-VPR containing the VP64 module and a multiple cloning site downstream of the *ACT1* promoter of the CIPACT plasmid (Fig. S1A).

**FIG 1:**
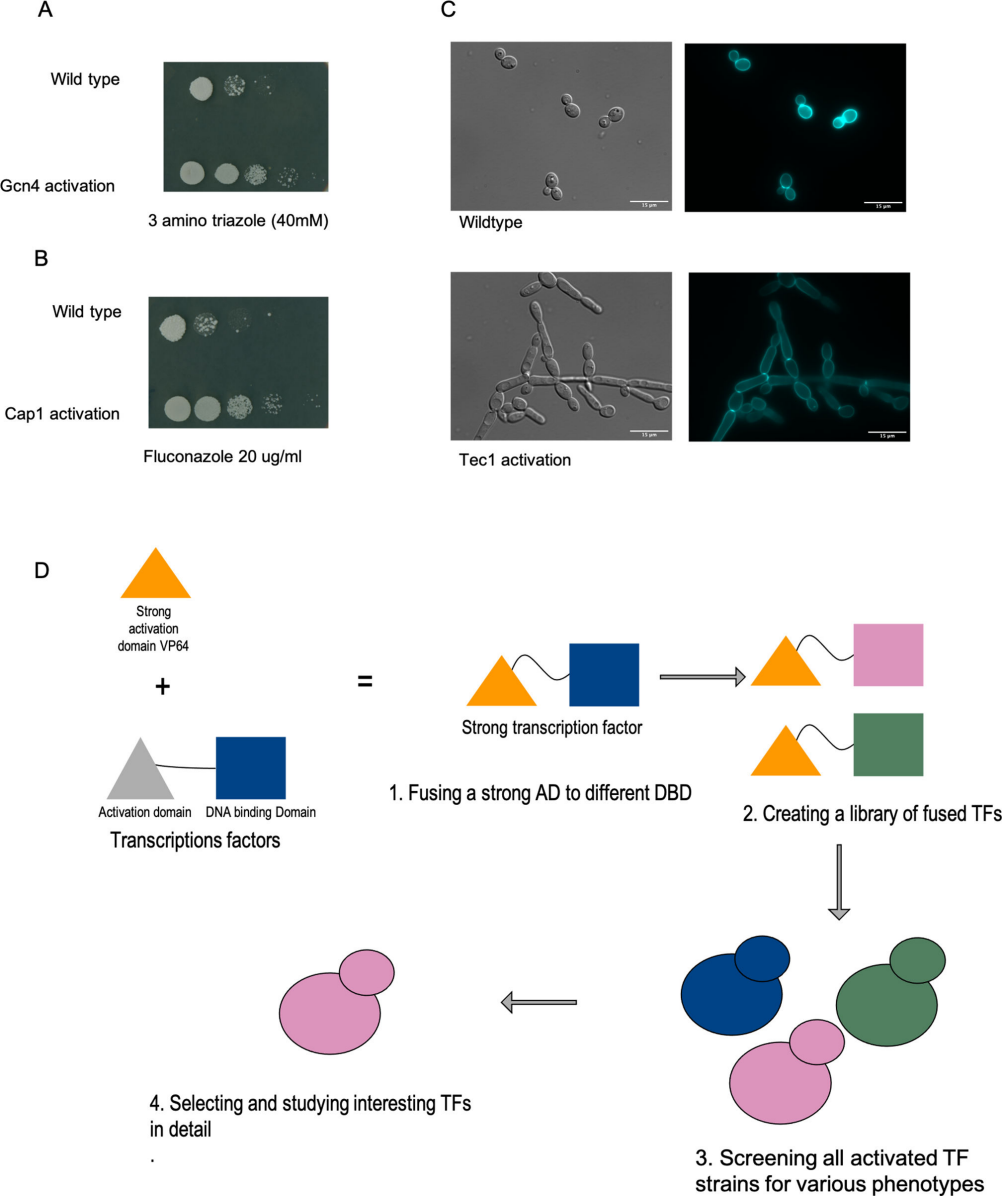
(A) VP64-Gcn4 chimeric transcription factor generates resistance to 3-amino-triazole (3AT). To construct the Gcn4-Vp64 fusion, we PCR amplified the Gcn4 DNA-binding domain and ligated it in the CIP-ACT-CYC plasmid in-frame with VP64 at the N terminus. After transforming this plasmid into *C. albicans*, we observed resistance to 3AT consistent with the VP64 module activating the transcription factor. (B) VP64-Tec1 TF triggers hyphal elongation in YPD media. Tec1 is a transcription factor implicated in the morphological switch from the *C. albicans* yeast form to the hyphal form. A construct containing the fusion of the N terminus of Tec1 to the VP64 module triggers elongated cellular growth. (C) VP64-Cap1 allows growth in SC media containing fluconazole. Cap1 is a poorly characterized transcription factor in *C. albicans* that gives resistance to azoles through Mdr1; activation of Cap1 upregulates MDR1 expression. Fusion of the VP64 module with Cap1 increased cellular tolerance to the azole fluconazole. (D) Schematic representation of the workflow involved in activating the transcription factors. Based on the success of the control constructs, we selected a set of transcription factors for fusion constructs and characterized the consequences of these fusions through phenotypic analysis.

To test the activation strategy, we chose three well-studied transcriptional factors—the bZIP TF Gcn4 (null mutant gives three amino-triazole sensitivity), the TEA/ATTS (homeo-domain) TF Tec1 (null mutant blocks hyphal development), and the leucine bZIP TF Cap1 (involved in fluconazole resistance). The Gcn4 construct generated resistance to three amino-triazole, consistent with upregulation of the Gcn4 target *HIS3* (Fig. 1A). The Tec1 construct triggered filamentation under yeast morphology growth conditions (Fig. 1B), and the Cap1 activation construct enhanced resistance to fluconazole (Fig. 1C).

We next selected 30 different TFs based on their phylogenetic uniqueness, their possible involvement in drug resistance, and their potential for functioning in rewired circuits. After generating these 30 TF-VP64 fusions, we first investigated their involvement in antifungal drug resistance (Fig. 1D). We selected three drugs for our preliminary screening—fluconazole, posaconazole, and amphotericin B. All three drugs target the cell membrane; fluconazole and posaconazole target lanosterol 14-alpha-demethylase (Erg11), an enzyme of the ergosterol pathway, whereas amphotericin B targets the end-product ergosterol. We identified two transcription factor fusions, encoded by C4_02500C_A and C6_00010W_A, that gave resistance to all three drugs. As the C4_02500C_A fusion created a higher level of resistance than the C6_00010W_A fusion, we prioritized C4_02500C_A for further study (Table 1).

**TABLE 1:** Screening of activated transcription factors in the presence of different drugs^*a*^

Transcription factor	Fluconazole	Posaconazole	Amphotericin B	Caspofungin
Try3 Orf19.1971	−	−	+	−
*C5_04410C_A* Orf19.3928	−	−	−	−
C3_01220W_A Orf19.1729	+	++	−	−
C1_04510W_A Orf19.6845	++	++	+	+
C2_05640W_A Orf19.6874	−	−	−	−
Hap41 Orf19.740	−	−	+	−
Hap42 Orf19.1481	++	++	+	+
Try6 Orf19.6824	−	−	−	+++
C2_02530W_A Orf19.1577	−	−	−	−
C2_01420C_A Orf19.1447	+	+	++	−
C4_07150W_A Orf19.3088	+	+	+	+
Uga33 Orf19.7317	−	−	−	−
Nrg2 Orf19.6339	++	++	++	++
Ace2 Orf19.6124	−	−	−	−
C1_13440C_A Orf19.4972	−	−	−	−
Rfx1 Orf19.3865	−	−	+	++
Grf10 Orf19.4000	+	−	+	+++
C4_05680W_A Orf19.1253	+	+	−	++
CR_05880W_A Orf19.6626	++	++	+	−
C2_08950W_A Orf19.211	+	+	−	+
C5_04280C_A Orf19.3912	−	−	−	−
**C4_02500C_A** **Orf19.2752 (Adr1)**	**+++**	**+++**	**+++**	**+**
C2_10660W_A Orf19.5343	+	+	+	+
C1_11690W_A Orf19.1150	+	+	−	−
C1_10020W_A Orf19.4869	−	−	+	++
C4_05880W_A Orf19.1275	−	−	−	−
Try4 Orf19.5975	−	−	−	+++
C4_05880W_A Orf19.1275	−	−	−	−
Met4 Orf19.5312	−	−	−	+
Gcn4	+	+	+	−
Cap1 (control)	+++	+++	++	+
Tec1 (control)	++	++	+	+

^*a*^ The optical densities are represented by plus signs (+), where + indicates growth equivalent to that of the positive control, ++ indicates a higher OD growth than + and +++ higher than ++ (−<+<++<+++ OD at 600 nm after 24 h) whereas - indicates no growth. Results focused on in this paper are in bold.

### Activation of C4_02500C_A confers multi-drug resistance

We subsequently tested whether the VP64 fusion to Orf19.2752 (C4_02500C_A) could trigger resistance to a variety of drugs—fluconazole, posaconazole, terbinafine, nystatin, caspofungin, anidulafungin, and amphotericin B (Fig. S1A and B). The fusion of C4_02500C_A to VP64 increases the minimum inhibitory concentration (MIC) as well as the minimal fungicidal concentration of fluconazole, amphotericin B, and terbinafine (Fig. S2). It also increased the MIC for these drugs, as well as for posaconazole and nystatin (Fig. S2B), by more than threefold. However, for the drugs caspofungin and anidulafungin that target the cell wall, there was no change in the MIC or growth rate for the activated strain relative to the control. Thus, activation of C4_02500C_A did not cause general drug resistance but did seem effective in generating resistance to cell-membrane-targeting drugs.

### Orf19.2752 (C4_02500C_A) is an ortholog of *S. cerevisiae* Adr1

Because TFs are frequently conserved across species, we searched for orthologs of the *C. albicans C4_02500C_A* gene. We found it to be highly similar to the *S. cerevisiae ADR1* gene; the two proteins have about 50% sequence identity, and the N-terminal DNA-binding domain is highly conserved between them (Fig. S2C).

In *S. cerevisiae*, Adr1, acting through a conserved binding motif 5′RCCCCM3′, is required for transcriptional regulation of ethanol, glycerol, and fatty acid utilization (27, 28). Due to the highly conserved DNA-binding domains of the two orthologs, we searched for this ScAdr1-binding motif upstream of *C. albicans* ORFs. We found 221 genes with this motif in their predicted promoter regions using Meme-suite software, as described in the Materials and Methods. Of the genes with this promoter motif, a significant number (one-tenth, or 20 genes) were implicated in ergosterol biosynthesis (Data File S1), while a further one quarter (52 genes) were categorized as having an unknown function. However, in contrast to the situation in *S. cerevisiae*, this motif is not enriched in ethanol, glycerol, and fatty acid metabolism genes in *C. albicans*. Because of the large number of motif-containing genes in the pathway of sterol biosynthesis, it appeared that CaAdr1 might instead be linked to sterol production.

### Adr1 DNA-binding motif

**FIG 2:**
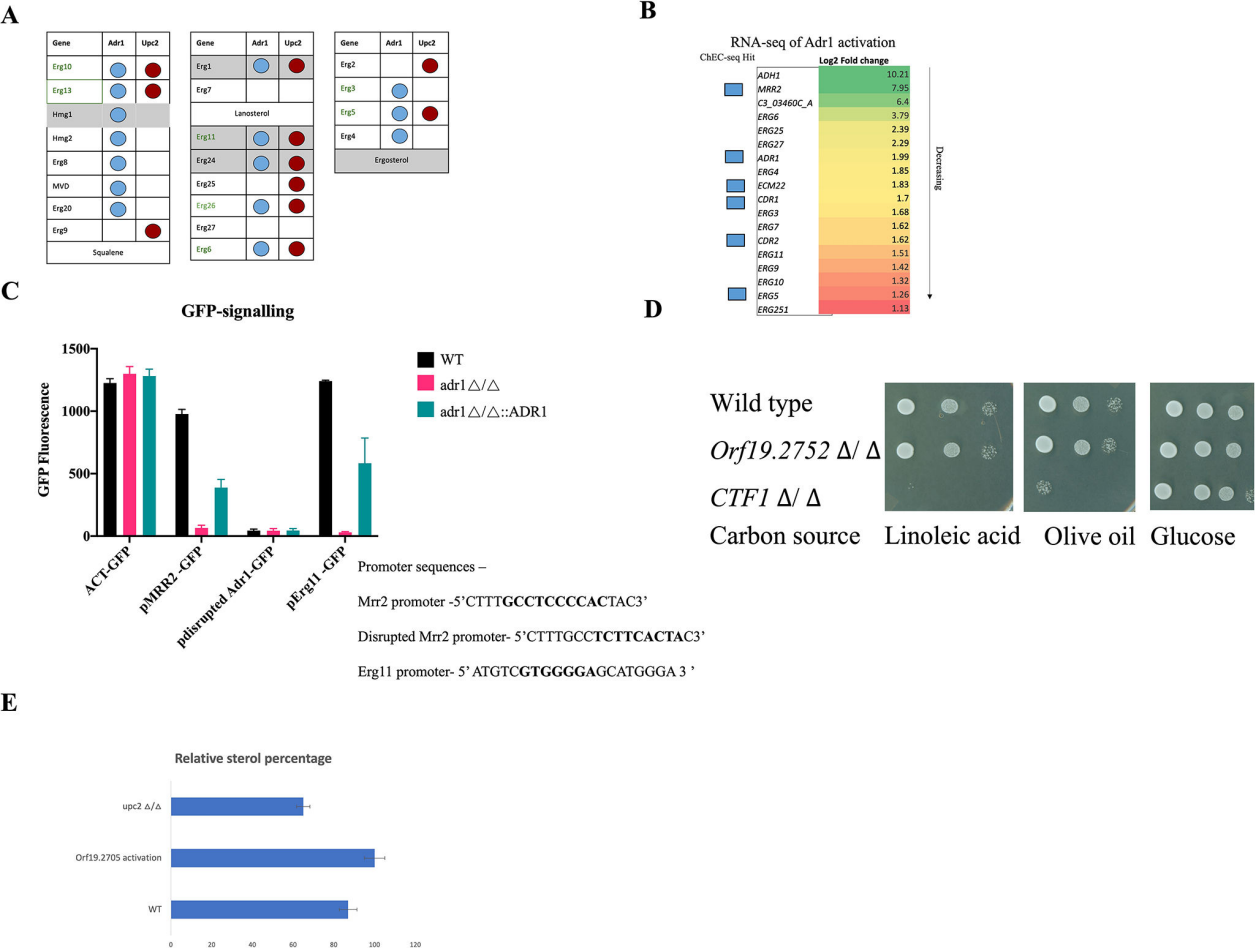
(A) The presence of the Adr1 motif upstream of ergosterol genes. The presence of the candidate Adr1 motif is represented by a blue circle. We found that most of the ergosterol genes have the candidate Adr1 motif 5′’NRCCCCM3′ in their promoter regions. (B) The transcriptomic profile of selected genes in the Orf19.2752-VP64 fusion strain shows upregulation of ergosterol genes. After activation of the Adr1 transcription factor, we did an RNAseq comparison of the activated strain and the wild type. We identified various ergosterol pathway genes that were upregulated and observed high expression of the genes for the transcription factor Mrr2 and the alcohol dehydrogenase Adh1. The full gene set is found in Fig. S2 along with the FPMK values. We also confirmed the binding of Adr1 by performing ChEC-Seq analysis; significantly bound genes are noted. (C) GFP reporter signaling. We measured, in both adr1Δ/Δ and wild-type strains, GFP driven by the ACT1 promoter, the ERG11 promoter with the potential Adr1-binding site 5′NRCCCCM3′, the MRR2 promoter with the potential Adr1-binding site 5′NRCCCCM3′, and disrupted the MRR2 promoter lacking the 5′NRCCCCM3′-binding site. We constructed GFP in the CIPACT-CYC plasmid by PCR and homology, replaced the ACT1 promoter with the ERG11 or MRR2 promoters, and disrupted Mrr2 in the wild-type strain CAI4 and the adr1 deletion strain as described previously. We quantified the GFP signaling using a BioTek Cytation 5. (D) Deletion of ADR1 (ORF19.2752). We deleted the ADR1 gene and checked the resulting strain for fatty acid, glycerol, and alcohol utilization. We found that it does not have any effect on growth on a fatty acid substrate, while a CTF1 deletion strain shows impaired growth on either linoleic acid or olive oil as a substrate. (E) Sterol estimation of wild-type and the Orf19.2752-activated strain. We isolated the sterols from overnight-grown *C. albicans* by the pargolol-hexane extraction method. The extracted sterols showed a four-peak spectral absorption pattern characteristic of ergosterol and 24(28)-DHE. The activated strain showed an approximately twofold increase in measured sterols.

We investigated potential direct 5′NRCCCCM3′ binding using ChEC-seq analysis. We fused the MNase cassette to Adr1 and used the calcium-activated nuclease to identify potential binding targets for Adr1. These results identified direct binding to several genes with the 5′NRCCCCM3′ motif in their promoters, including Mrr2, Adh1, Ecm22, Erg5, Erg28, Cdr1, and Adr1 itself. These genes were also upregulated in the Adr1-activation RNA-seq data set, suggesting that Adr1 may control 5′NRCCCCM3′ motif-bearing genes and thus be directly involved in transcriptional regulation of the ergosterol biosynthesis pathway (Fig. 2B and C). As well, the transcription factor Mrr2 was dramatically upregulated; this could help explain the observed multi-drug resistance phenotype, as Mrr2 acts to upregulate the *CDR* transporter-encoding genes, and our RNA-seq analysis shows that both *CDR1* and *CDR2* are more highly expressed in the Adr1-VP64 fusion strain than in the control (Fig. 2B). To establish if the fluconazole resistance is a direct result of this upregulation of Mrr2, we deleted *MRR2* in the Adr1-activated strain. Deletion of *MRR2* had essentially no effect on fluconazole resistance driven by activation of Adr1, suggesting that the upregulation of the Mrr2 TF was in fact not critical in creating the azole-resistance phenotype (Fig. S1B). Similarly, amphotericin B resistance was not affected by *MRR2* deletion. However, the deletion did impact the terbinafine resistance, suggesting that the observed allylamine resistance could be mediated through Mrr2 upregulation (Fig. S1D).

To further validate that Adr1 binds to the promoter sequence 5′NRCCCCM3′, we put a GFP reporter construct under control of the *ERG 11* promoter, the *MRR2* promoter, and, to serve as a control, the *ACT1* promoter. We introduced these constructs into both wild-type and *adr1* deletion strains. We found that the *adr1* deletion strain generated a much weaker GFP signal from the *ERG11* and *MRR2* promoters than the signals from the wild-type strain, while the expression of the *ACT1* construct did not differ between the two strains (Fig. 2D). These results supported the requirement of Adr1 for the promoter function of *ERG11* and *MRR2*. We then disrupted the NRCCCCM sequence in the *ERG11* and *MRR2* GFP reporter constructs and saw background GFP signals from both the wild-type and *adr1* deletion strains. This confirms that NRCCCCM is the sequence through which the Adr1 transcription factor functions (Fig. 2D).

### CaAdr1 influences sterol metabolism

Azole drugs target Erg11 of the ergosterol pathway in *C. albicans* (29)*,* and upregulation of Erg11 is one of the known mechanisms for drug resistance against fluconazole (30, 31). To test if the *ADR1* gene of *C. albicans* was involved in sterol metabolism, we deleted the gene and checked the consequences of loss of function; consistent with a role in sterol biosynthesis, *ADR1* deletion causes slight sensitivity to cell membrane targeting drugs like fluconazole (Fig. S1C and D; Fig. S2A). The complementation of *adr1* with the native protein or the Vp64-activated version restored the drug sensitivity (Fig. S1C). However, unlike the situation with *ScADR1*, the deletion of *CaADR1* did not block growth on fatty acid substrates (Fig. 2). By contrast, deletion of the gene for the transcription factor Ctf1, identified as a regulator of fatty acid metabolism genes in the pathogen (32), completely blocked *C. albicans* growth on linoleic acid and severely compromised growth on olive oil (Fig. 2D). This suggests that in *C. albicans*, Ctf1 is controlling fatty acid utilization, while Adr1 is not involved. In *S. cerevisiae*, ScAdr1 was found to be haplo-insufficient for ethanol, glycerol, and fatty acid metabolism. Similarly, in *C*. *albicans*, CaAdr1 is haplo-insufficient for fluconazole resistance, as the heterozygote showed sensitivity to fluconazole relative to the WT but was clearly more resistant than the homozygous null (Fig. S1C).

We directly checked the sterol content of the Adr1-activated and wild-type strains by extracting sterols with the organic solvents pargolol and hexane, followed by spectrophotometric assessment. Activated CaAdr1 enhances the production of ergosterol (Fig. 2E). We also directly assessed the transcriptional consequences of Adr1 activation through RNAseq analysis. *ADH1*, which encodes the alcohol dehydrogenase that oxidizes ethanol to acetaldehyde, was the most highly upregulated gene in our profile, and intriguingly, the orthologous gene in *S*. *cerevisiae* is a direct target of ScAdr1 (33).

### Adr1 and Upc2 roles in azole response

**FIG 3:**
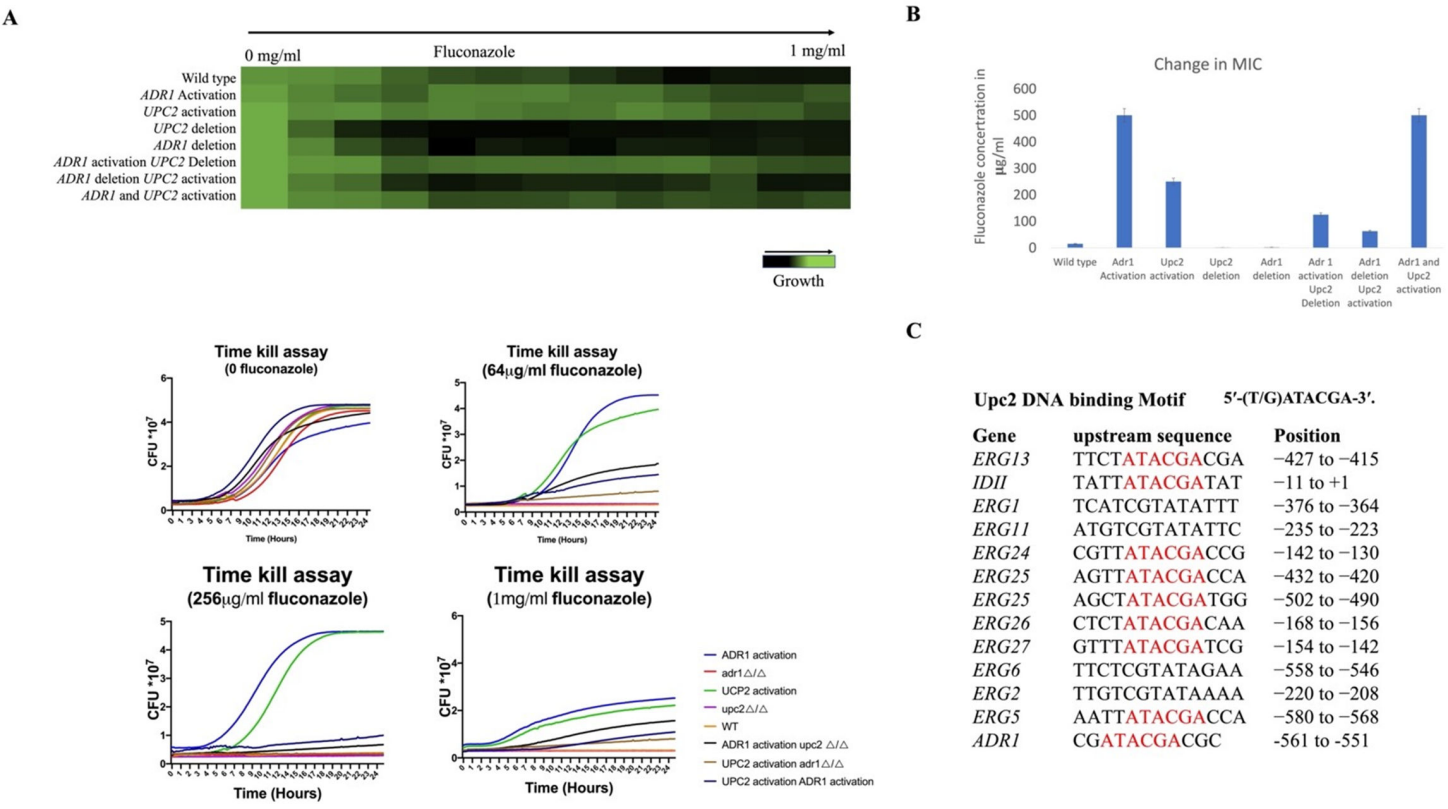
(**A**) Adr1 and Upc2 influence the response to fluconazole. Fluconazole MIC assay of ADR1 deletion, ADR1 activation, ADR1 activation in UPC2 deletion, ADR1 deletion in UPC2 activation and double activation, followed by the time kill assay. (**B**) Fold change in MIC from Adr1 and Upc2 activation and deletion. Graphical representation of the change in minimum inhibitory concentrations of various drugs in the Orf19.2752-activated strain. (**C**) Upc2 DNA-binding motif upstream of various regulons, including ADR1. Upstream regions of genes, including the transcription factors Adr1, Upc2, Mrr2, and Mrr1, as well as the structural genes for the ergosterol biosynthesis pathway, were examined for candidate TF-binding motifs. We identified a potential Upc2-binding motif upstream of the ADR1 gene.

Consistent with the presence of the candidate Adr1-binding motif in their promoters, we found that the expression level of most of the ergosterol pathway genes, including Erg11, was upregulated by the activated Adr1 construct. This increase in ergosterol pathway gene expression was, however, not associated with upregulation of the classic ergosterol biosynthesis pathway transcription factor Upc2, which functions as a key regulator of the pathway in both *S. cerevisiae* and *C. albicans* (34 – 36). Therefore, it appears that Adr1 activation of the *C. albicans* ergosterol pathway genes is likely direct (Table S1), and thus the fluconazole resistance generated by the Adr1VP64 fusion protein may be due to the generalized increase in the expression of these ergosterol biosynthetic pathway genes. We then asked whether the fluconazole resistance generated by Adr1 activation was fully independent of Upc2. First, we compared fluconazole resistance levels in strains with Adr1 activated and with Upc2 activated, as well as in strains with deleted *UPC2* or *ADR1*. As shown in Fig. 3A and B, both Upc2 activation and Adr1 activation gave similarly high levels of resistance to fluconazole, while both Adr1 deletion and Upc2 deletion conferred sensitivity to fluconazole, with the Upc2 deletion strain being somewhat more sensitive. Second, we assessed the resistance to fluconazole of the doubly activated strain; in this case, the strain grew poorly in the absence of the drug but was resistant to fluconazole at similar levels to that of the single-activated mutants (Fig. 3A and B). Finally, we investigated the behavior of cells with activated Adr1 that lacked functional Upc2 and cells with activated Upc2 that lacked Adr1. We observed that loss of Upc2 had essentially no effect on fluconazole resistance caused by activation of Adr1, suggesting that the effect of Adr1 on drug resistance is independent of Upc2, while loss of Adr1 significantly compromised fluconazole resistance caused by activation of Upc2 (Fig. 3A and B). This is consistent with part of the effect of Upc2 activation on azole resistance working through Adr1. We checked the upstream sequences of the *UPC2* and *ADR1* genes for regulatory motifs and found that the promoter sequence of the *ADR1* gene contains a potential *UPC2* motif (Fig. 3C), consistent with Adr1 being part of the Upc2 regulon, while the *UPC2* promoter lacks any potential Adr1-binding motif.

### Phylogenetic analysis

We examined a phylogenetic profile of the Ascomycota and characterized the upstream sequences of all ergosterol biosynthesis genes for the regulatory motifs associated with both the Adr1 and the Upc2 transcription factors. In *C. albicans*, our data suggest that Adr1 works together with Upc2 to control the ergosterol biosynthesis pathway, with Adr1 also controlling the expression of genes such as *ADH1,* which encodes the alcohol dehydrogenase that oxidizes ethanol to acetaldehyde, and *MRR2,* which encodes a stress-responsive transcription factor. The more basal filamentous fungi also have candidate binding motifs for both Adr1 and Upc2, the promoters of the ergosterol biosynthesis genes. However, the presence of the Adr1 motif in the promoter of *MRR2* is very specific to the CTG clade species (Data file S1).

In the evolutionary trajectory leading to *S. cerevisiae*, it appears that the Adr1 TF was repurposed to control alcohol, fatty acid, and glycerol utilization, taking over from Ctf1 orthologs that do the job in the filamentous fungi and the CTG clade species. Based on the search of promoter motifs, we identified that the Upc2 binding motif is found in the promoters of ergosterol pathway genes throughout the fungi, along with the Adr1 motif. But after *Candida guilliermondii* in the phylogeny, the Adr1 motif signal weakens, leaving only Upc2 with a strong signal associated with the ergosterol pathway. The Adr1-binding motif signal connecting the ergosterol pathway genes in the CTG clade appears to be gradually transferred to genes involved in the control of alcohol and fatty acid utilization in *S. cerevisiae* and its relatives (Data file S1).

## DISCUSSION

One of the common approaches to investigating the regulatory networks controlling drug resistance in fungal pathogens is through comparison with the *S. cerevisiae* circuits; this approach has led to the discovery of many TFs responsible for drug resistance in both *S. cerevisiae* and *C. albicans*, including Fcr1, Ndt80, Mrr1, and Upc2 (34, 37 – 40). However, these two fungi diverged as long ago as 300 million years, and for species that diverged by such an evolutionary distance, most of the DNA-binding patterns of a given regulator in one species are unlikely to be preserved in the other species (20). Overall, genome-wide correlations converge on about 10% overlap for species with this level of divergence (20), and therefore, there is a significant probability that many of the TFs responsible for drug resistance will be different between *C. albicans* and budding yeast. To identify candidate TFs with *Candida*-specific roles in drug resistance, we selected a set of TF-encoding genes that were either not found in *S*. *cerevisiae* or predicted to have potentially changed function between the two species. We identified 30 such TFs and activated them to identify potential roles in drug resistance (as well as other cellular processes) (Table 1). Among these transcription factors, Orf19.2752 activation resulted in clear resistance to a set of drugs generally targeting the cell membrane; activation of this transcription factor generated resistance to azoles, allylamines, and polyenes. Sequence comparisons established that *C4_02500C_A (ORF19.2752*) was the *C*. *albicans* ortholog of the *S. cerevisiae* gene *ADR1*, a gene not linked to drug resistance in budding yeast. These two transcription factors share a highly conserved DNA-binding domain.

In *S. cerevisiae*, Adr1 is involved in the transcriptional regulation of genes involved in the catabolism of ethanol, glycerol, and fatty acids (27, 28) and is proposed to act through a candidate DNA-binding motif, 5′NRCCCCM3′, in the promoter regions of these genes (41). Interestingly, in *C. albicans*, this same DNA-binding motif is enriched in the upstream regions of the ergosterol pathway genes, whereas it is generally absent from the promoters of the ethanol, glycerol, and fatty acid utilization genes of this pathogen. This suggests that Adr1 may have been rewired from the ergosterol biosynthesis pathway in other fungi to the metabolic utilization of ethanol, glycerol, and fatty acids in *S. cerevisiae*. Further investigation established that activation of CaAdr1 generated transcriptional upregulation of most of the ergosterol pathway genes in the pathogen. However, Upc2, the well-established regulator of the Erg-pathway genes in both *C. albicans* and *S. cerevisiae*, was not transcriptionally upregulated, suggesting that in *C. albicans*, Adr1 activation of the ergosterol pathway genes was not going through Upc2.

To further investigate the proposed Adr1 DNA-binding motif, we performed a ChEC-seq analysis (42, 43) and found that several drug-resistance-related genes, including Mrr2, Adh1, Ecm22, Erg5, Ddr48, Erg28, Cdr1, and Adr1, were both upregulated in the RNAseq analysis and were ChEC-seq hits. Previous *in-silico* analysis of a number of TFs had shown a weak but statistically significant overlap in the genes in *S. cerevisiae* and *C. albicans* containing the Adr1 motif in their promoters (44). This is consistent with our ChEC-seq analysis; while many genes have been rewired between the two species, some genes, like *ADH1*, are under Adr1 control in both species, suggesting that Adr1 might have multiple roles in both fungi. However, the bulk of the circuit of ethanol, fatty acid, and glycerol metabolism controlled by Adr1 in *S. cerevisiae* is under the control of Ctf1 in *C. albicans* (32), as Adr1 deletion did not affect growth on substrates like olive oil and linoleic acid, whereas Ctf1 deletion gives a clear auxotrophy. Apart from Adh1, in our RNA-seq as well as CheC-seq results, we found transporter, metabolism, and stress-responsive genes, including oxidative stress. Previously, it was reported that amphotericin B resistance might be due to upregulation of oxidative stress genes (45).

These data underline the multiple circuit restructurings involved in the control of these pathways in different fungi. In *S*. *cerevisiae*, Cat8 and Adr1 both appear to have been rewired to connect to the module controlling alcohol, acetic acid, and fatty acid utilization, Adr1 from the ergosterol circuit, and Cat8 from gluconeogenesis (ScSip4). Another event is the disappearance of the Ctf1 TF from the *S*. *cerevisiae* genome, as there is no apparent ortholog of Ctf1 in *S*. *cerevisiae*. This loss could be facilitated by the transfer of the Ctf1 regulon to Adr1 control in *S. cerevisiae*. In *S*. *cerevisiae*, Upc2 gains a paralog (Emc22) and apparently unique control over the ergosterol pathway (46, 47).

In *C. albicans*, Adr1 activation causes upregulation of many ergosterol pathway members, including Erg11 (target of azoles), Erg1 (target of allylamines), Erg2 (target of morpholines), HMG1/2 (target of statins), and causes increases in ergosterol itself (target of polyenes), which has the potential to generate multi-drug resistance. The activation of Adr1 dramatically enhances the MIC of fluconazole, amphotericin B, terbinafine, and statins. We created a series of strains to determine how Upc2 and Adr1 are influencing the ergosterol pathway. Activation of either Upc2 or Adr1 enhanced azole resistance, while deletion of either gene created azole sensitivity. Activation of both TFs at the same time caused poor growth, perhaps due to the disturbed circuits or due to the activation of an Erg3-driven side branch of the pathway generating the toxic 14-methyl-ergosta-8,24(28)-dienol. However, the relative resistance to azoles remained similar to the individually activated strains, suggesting that the actions of the two TFs are not additive or synergistic. Another known stress associated with ergosterol is hypoxia, and therefore, we tested both Adr1 activation and *adr1* deletion under hypoxia conditions (48, 49). We did not find any changes in the *adr1* deletion strain under hypoxia, while the upregulated allele somewhat improved growth (Fig. S2F). Previously, the *upc2* deletion strain has been reported to show a significant growth defect under hypoxia (50), so this distinction will need further investigation.

The fluconazole resistance caused by activation of Upc2 is significantly dependent on the presence of Adr1, but loss of Upc2 had very little effect on the resistance profile of strains with activated Adr1. In addition, there is a potential Upc2 DNA-binding site in the promoter region of *ADR1*, while *UPC2* has no candidate site for Adr1. These results are consistent with Upc2 serving as a master regulator of ergosterol biosynthesis, controlling ERG gene expression both directly and through regulation of the Adr1 TF, which also serves as an activator of ERG gene expression. In the absence of Upc2, Adr1 is sufficient to ensure ERG expression, although response to azole drugs is compromised by loss of either TF. A strain containing deletions of both *adr1* and *upc2* was very slow growing and also very sensitive to fluconazole.

Among the highly upregulated genes resulting from Adr1 activation is the gene encoding Mrr2, itself a TF involved in the expression of the multi-drug-resistance-regulating membrane transporter Cdr1. However, even though *CDR1* expression was somewhat upregulated in the Adr1-activated strain, the Mrr2 upregulation did not seem critical for the observed fluconazole resistance because deletion of *MRR2* in the Adr1-activated strain did not compromise this resistance.

While it appears that in filamentous fungi and the CTG clade species Adr1 is linked to ergosterol biosynthesis, in the Saccharomycotina, it has been shifted to control the pathway for ethanol, glycerol, and fatty acid utilization (51, 52), replacing Ctf1 that controls the process in the non-Saccharomycotina species. This transfer appears so complete that the Ctf1 factor has been lost in the Saccharomycotina. Intriguingly, throughout this transition, Adr1 has maintained a role in the control of the expression of the alcohol dehydrogenase catalyzing the oxidation of ethanol to acetaldehyde (*ADH1* in *C. albicans*, *ADH2* in *S. cerevisiae*). To deal with ethanol toxicity, in *S*. *cerevisiae*, ethanol is modified into unsaturated lipids and ergosterol (51). The rewiring of Adr1 from the ergosterol pathway to the ethanol utilization process (41, 52) may have been driven by the shift to the Crabtree-positive lifestyle of *S. cerevisiae*, requiring the ability to both tolerate and process high concentrations of ethanol.

### Conclusion

**FIG 4:**
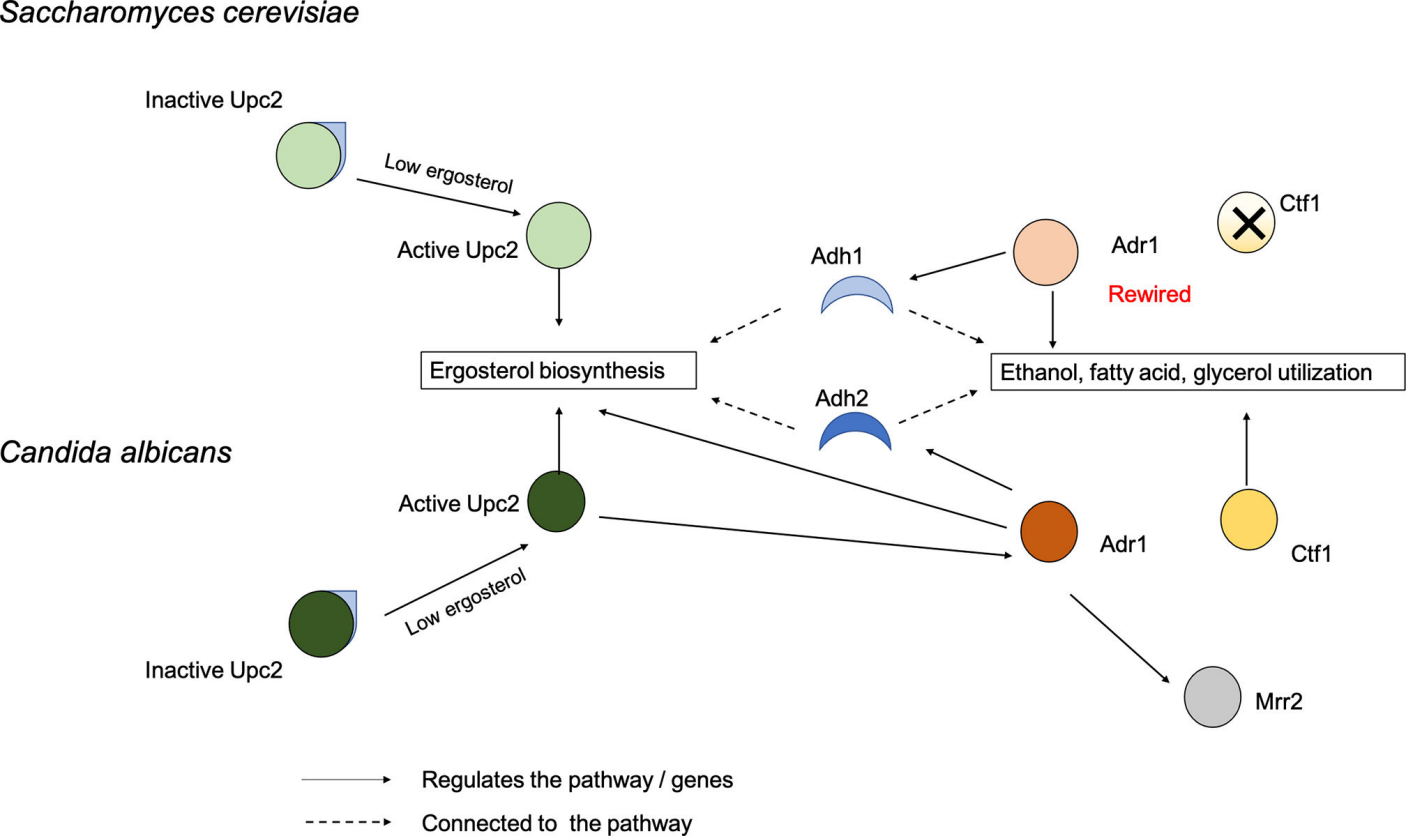
Proposed model of the multiple rewiring of transcription factors between *S. cerevisiae* and *C. albicans*, along with coordination of Upc2, Adr1, and Mrr2 during the presence of azoles or depletion of ergosterol, leading to drug resistance in *C. albicans*. In both *S. cerevisiae* and *C. albicans*, the Upc2 transcription factor binds to ergosterol and remains inactive in the cytosol. Depletion of ergosterol changes Upc2 to an active form, which enters the nucleus and initiates the transcription of the genes for ergosterol biosynthesis. In *C. albicans*, activated Upc2 also triggers expression of the Adr1 transcription factor, which further serves to direct expression of ergosterol biosynthesis genes as well as the alcohol metabolism gene Adh1 and the Mrr2 transcription factor. In *S. cerevisiae*, Adr1 has been rewired to control other parts of the alcohol utilization circuit in addition to alcohol dehydrogenase, as well as both fatty acid and glycerol utilization circuits.

Sterol biosynthesis is critical for fungal biogenesis and a target for many antifungal drugs. We have identified the TF Adr1 as a key regulator of sterol biosynthesis in the fungal pathogen *C. albicans*, where it works in concert with the zinc cluster TF Upc2. We suggest that Upc2, when bound to ergosterol, remains inactive in the cytoplasm but is activated when there is depletion of ergosterol, whether by environmental factors or due to the presence of drugs targeting the ergosterol pathway. Activated Upc2 goes to the nucleus and turns on key players of the ergosterol biosynthetic pathway as well as the TFs Mrr1 and Adr1. Adr1 aids in regulating the ergosterol pathway genes and turns on the TF Mrr2. Thus, activation of Upc2 and Adr1 leads to the coordinated expression of the ergosterol biosynthesis pathway, as well as the activation of the phospholipid transporters Cdr1 and Cdr2, which can also function to export antifungal drugs (Fig. 4).

## MATERIALS AND METHODS

### Bioinformatics analysis

Sequences of genes Ca*ADR1* and *ScADR1* were obtained from the Candida Genome Database (CGD; http://www.candidagenome.org/) and the Saccharomyces Genome Database (https://www.yeastgenome.org/). Gene orthogroup assignments for all predicted protein-coding genes across 23 ascomycete fungal genomes were obtained from the Fungal Orthogroups Repository (53) and maintained by the Broad Institute.

DNA sequence motifs were identified using the web-based motif-detection algorithm MEME ( ht tp://meme.sdsc.edu/meme/intro.html ) (54). For more stringent motif identification, we used MAST hits with an *E*-value of <50. An *E*-value of 500 corresponds roughly to a *P*-value of 0.08 in our analysis, and an *E*-value of 50 roughly corresponds to a *P*-value of 0.008. We also used AME ( ht tp://meme-suite.org/tools/meme ), which identifies known motifs throughout the *Candida* upstream sequences.

Protein domains and linear motifs were detected from each individual TF protein sequence using INTERPROSCAN, PFAM, and ELM motif definitions. For ChEC-seq analysis, we used Integrative Genomics Viewer (IGV) (https://igv.org) and MEME-ChIP software.

### Strains and culture conditions

For general growth and maintenance of the strains, the cells were cultured in fresh YPD medium [1% (wt/vol) yeast extract, 2% (wt/vol) Bacto peptone, 2% (wt/vol) dextrose, and 80 mg/L uridine with the addition of 2% (wt/vol) agar for solid medium] at 30°C. For drug assays, we used synthetic dextrose (SD) medium [0.67% (wt/vol) yeast nitrogen base, 0.15% (wt/vol) amino acid mix, 0.1% (wt/vol) uridine, 2% (wt/vol) dextrose, and 2% (wt/vol) agar for solid media] along with the various concentrations of drugs in liquid and solid media.

### Gene knockouts using CRISPR

All *C. albicans* mutants were constructed in the wild-type strain CAI4. The protocol used for the CRISPR-mediated knockout of *ADR1*, *CTF1*, and *UPC2* was adapted from (55); we used *URA3* replacements in our study. CRISPR-mediated knockouts used the lithium acetate method of transformation with the modification of growing transformants overnight in liquid YPD at room temperature after removing the lithium acetate-PEG. *C. albicans* transformants were selected on SD-URA plates.

### Activation of transcription factors

For the activation module, the *ACT1* promoter and VP64 were amplified by PCR, and homology was created by primer extension such that there is an Mlu I site between *ACT1* and VP64. This ligated CIPACT-VP64 plasmid was transformed into *Escherichia coli* using the calcium chloride method. High-throughput equipment at the Concordia Genome Foundry, the Biomek FXP Automated Workstation, otherwise known as the Biomek FXP liquid handler, was used to insert different DNA-binding domains to create and later screen this library.

Plasmids extracted from colonies that were determined to have the guide sequence successfully cloned were then used to transform *C. albicans* using a lithium acetate transformation protocol. pCIPACT1 was linearized by StuI-HF digestion, and 1–2 μg of the linearized plasmid was used in the transformation. *C. albicans* transformants were selected on SD-URA plates.

### RNA seq analysis

The CaAdr1 and SC5143 strains were grown in SC media overnight at 30°C, diluted to an OD600 of 0.1 in YPD at 30°C, and then grown to an OD600 of approximately 1.0 on a 220-rpm shaker. Total RNA was extracted from two biological replicates using the Qiagen RNeasy Minikit protocol, and RNA quality and quantity were determined using an Agilent bioanalyzer. Paired-end sequencing (150 bp) of extracted RNA samples was carried out at the Quebec Genome Innovation Center located at McGill University using an Illumina miSEQ sequencing platform. Raw reads were pre-processed with the sequence-grooming tool cutadapt version 0.4.1 (56) with the following quality trimming and filtering parameters: `--phred33 --length 36 -q 5 --stringency 1 -e 0.1`. Each set of paired-end reads was mapped against the *C. albicans* SC5314 haplotype A, version A22, downloaded from the CGD ( http://www.candidagenome.org/) using HISAT2 version 2.0.4. SAM tools were then used to sort and convert SAM files. The read alignments and *C. albicans* SC5314 genome annotations were provided as input into StringTie v1.3.3 (57), which returned gene abundances for each sample. Raw and processed data have been deposited in NCBI’s Gene Expression Omnibus (58).

### Sterol quantification

The cells were grown overnight (16 h) at 30°C and harvested by centrifugation. Non-treated cells were maintained separately and considered controls. The cell pellets were washed twice with sterile distilled water. We followed the same method that has been described in reference (59) with slight modifications. Cell pellets were resuspended in 2.5 mL methanol, 1.5 mL potassium hydroxide [60% (wt/vol)], and 1 mL methanol-dissolved pyrogallol [0.5% (wt/vol)] and heated at 90°C for 2 h. The cell extracts were cooled, and then sterols were extracted with two rounds of treatment with 5 mL of hexane. The extracted sterols indicated a four-peak spectral absorption pattern produced by ergosterol and 24(28)-dehydroergosterol [24(28)-DHE] spectrophotometrically (DU530 life science UV spectrophotometer). Both ergosterol and 24(28)-DHE absorb at 281.5 nm, whereas only 24(28)-DHE absorbs at 230 nm. The ergosterol content is determined by subtracting the amount of 24(28)-DHE (calculated from the A230) from the total ergosterol plus the 24(28)-DHE content (calculated from the A281.5). Ergosterol content was calculated as a percentage of the wet weight of the cells with the following equations: % ergosterol + % 24(28)-DHE [(A281.5/290) × *F*] / pellet weight, % 24(28)-DHE − [(A230/518) − *F*] / pellet weight, and % ergosterol = [% ergosterol + % 24(28)-DHE] – [% 24(28)-DHE], where *F* is the factor for dilution in petroleum ether and 290 and 518 are the *E*-values (in percent per centimeter) determined for crystalline ergosterol and 24(28)-DHE, respectively.

### ChEC-seq analysis

To perform the ChEC-seq analysis, we followed reference (43). We constructed the Adr1-MNase strain and grew overnight cultures of *C. albicans* Adr1-MNase-tagged and free MNase strains that were then diluted to a starting OD600 of 0.1 in 50 mL YPD medium and grown at 30°C to an OD600 of 0.7 to 0.8. Cells were washed three times with 1 mL buffer A [15 mM Tris (pH 7.5), 80 mM KCl, 0.1 mM EGTA, 0.2 mM spermine, 0.5 mM spermidine, one tablet of Roche complete EDTA-free mini protease inhibitors, 1 mM phenylmethylsulfonyl fluoride], resuspended in 800 µL buffer A containing 0.1% digitonin (Sigma), and permeabilized for 10 min at 30°C with shaking. MNase digestions were performed by adding CaCl_2_ to a final concentration of 5 mM and incubating for different time points at 30°C. At each time point, a total of 200 µL aliquots of the ChEC digestions were transferred to a tube containing 50 µL of 250 mM EGTA to quench MNase digestion. DNA was extracted using a MasterPure yeast DNA purification kit (Epicentre, MPY80200). ChEC DNA was subjected to size selection using the Pippin Prep (SageScience) size selection system with a 2% agarose gel cassette, allowing the removal of multi-kilobase genomic DNA fragments and the enrichment of 100 to 400 bp DNA fragments.

For the GFP signaling quantification, we introduced GFP in the CIPACT-CYC plasmid by PCR, and homology was created by primer extension for enzymes XmaI and MluI. Furthermore, we replaced the *ACT1* promoter with the *ERG11* or *MRR2* promoters and disrupted Mrr2 using Kpn1 and an enzyme. The ligation was done using T4 DNA ligase and was transformed into *E. coli* using the calcium chloride method. These plasmids were further transformed into the wild-type strain CAI4 and the *adr1* deletion strain as described previously. The GFP signals were quantified using a BioTek Cytation 5.

## SUPPLEMENTAL MATERIAL

Data File S1 mbio.01807-23-s0001.xlsx All the raw data and bioinformatic analysis.

Figure S1 mbio.01807-23-s0002.tif Supplemental information, including the construct for activation of non-zinc cluster transcription factors.

Figure S2 mbio.01807-23-s0003.tif Supplemental information, including information on Orf19.2752 and fluconazole, posaconazole, terbinafine, and amphotericin B resistance.

Supplemental legends mbio.01807-23-s0004.docx Legends for Fig. S1 and S2.

ASM does not own the copyrights to Supplemental Material that may be linked to, or accessed through, an article. The authors have granted ASM a non-exclusive, world-wide license to publish the Supplemental Material files. Please contact the corresponding author directly for reuse.
