# Supplementary figures and images for: The Adr1 transcription factor directs regulation of the ergosterol pathway and azole resistance in Candida albicans
Source: mBio. 2023 Oct 4;14(5):e01807-23. doi: 10.1128/mbio.01807-23 (PMC10653825; doi:10.1128/mbio.01807-23)

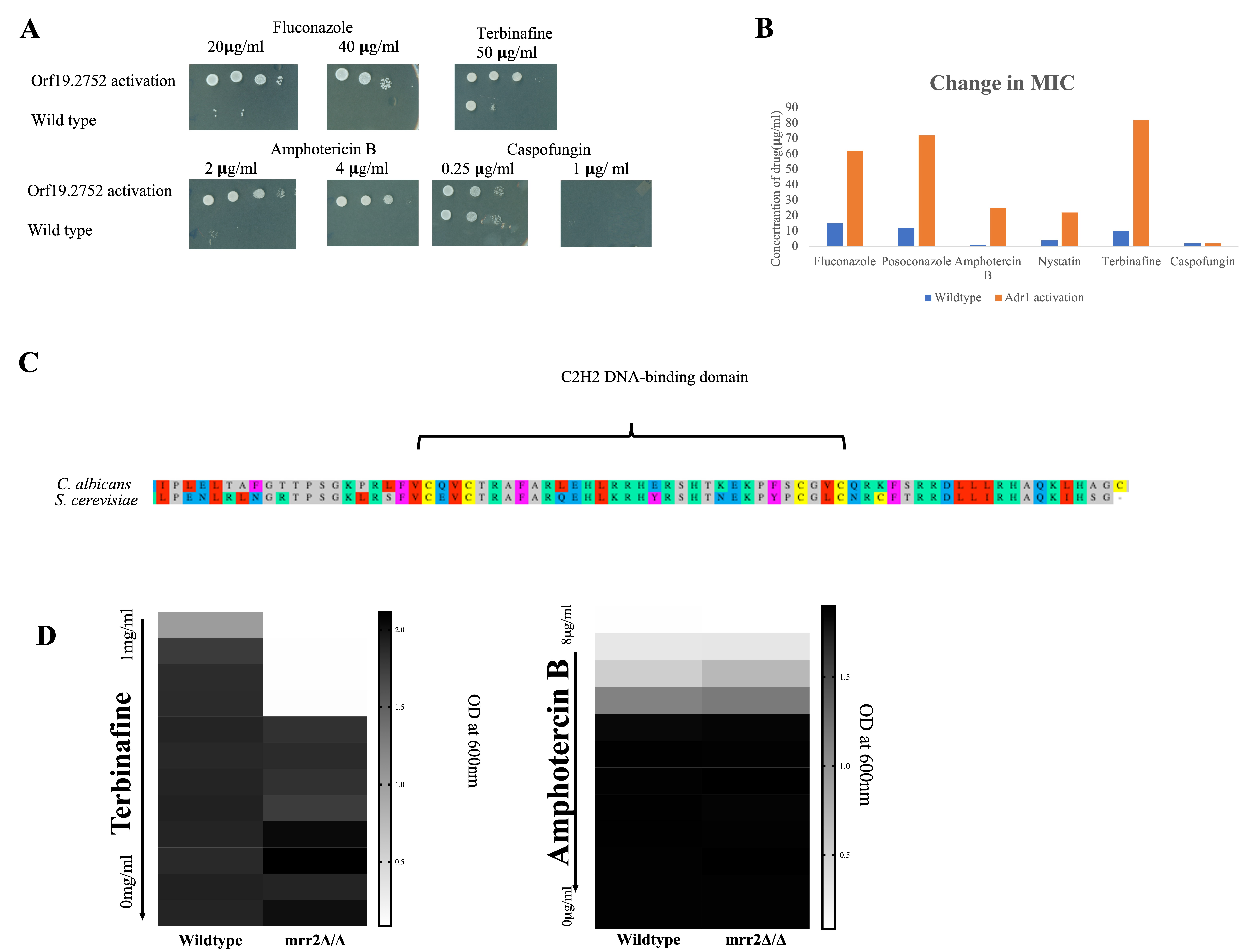

Supplement: Figure S2 — Supplemental information, including information on Orf19.2752 and fluconazole, posaconazole, terbinafine, and amphotericin B resistance. [file mbio.01807-23-s0003.tif]
