# Supplementary material for: The Adr1 transcription factor directs regulation of the ergosterol pathway and azole resistance in Candida albicans
Source: mBio. 2023 Oct 4;14(5):e01807-23. doi: 10.1128/mbio.01807-23 (PMC10653825; doi:10.1128/mbio.01807-23)
Supplement: Supplemental legends — Legends for Fig. S1 and S2. [file mbio.01807-23-s0004.docx]

***Fig. S1. (A) The construct for activation of non-zinc cluster transcription factors.***

***(B)* *The adr1Δ/Δ strain (ORF19.2752) showed fluconazole sensitivity. The heterozygous adr1Δ/+ shows better growth than adr1Δ/Δ in the presence of fluconazole.***

***(C)* *The mrr2 Δ/Δ in the Adr1 activated strain caused no changes in azole resistance.***

***(D) The graphical representation of the growth variation in presence of different concentrations of fluconazole in the adr1Δ/Δ, adr1Δ/Δ with complementation of hyperactivated ADR1(Adr1-VP64) allele, adr1Δ/Δ with complementation of the native ADR1 allele, Adr1 hyperactivation (Adr1-VP64) and wildtype.***

***(E)The graphical representation of the growth variation under Hypoxia in the adr1Δ/Δ , Adr1 hyperactivation (Adr1-VP64), Upc2Δ/Δ, Upc2 hyperactivation and wildtype.***

***Fig. S2. (A) Plate assay of SC agar with different drugs confirming the VP64 fusion of Orf19.2752 generates fluconazole, terbinafine and amphotericin B resistance.*** *We used minimal inhibitory concentration (MIC) and 2MIC concentrations to check if the fusion construct creates resistance to the drugs.*

***(B) Activated Orf19.2752 caused fluconazole, posaconazole, terbinafine and amphotericin B resistance compared to wildtype.*** *Graphical representation of the change in minimal inhibitory concentrations of the Orf19.2752 activated strain in liquid SC media containing the various drugs. After 24 hrs cells were checked for residual growth activity on Sc media.*

***(C) Sequence alignment shows that Orf19.2752 is similar to the Adr1 protein of S. cerevisiae****. Blast alignment of the zinc cluster DNA binding domain of the Adr1 transcription factor in S. cerevisiae with that of Orf19.2752 in C. albicans.*

***(D)The graphical representation of the growth variation in presence of different concentrations of terbinafine***

***and amphotericin B in the mrr21Δ/Δ and wildtype.***
